# Small animal PET with spontaneous inhalation of ^15^O-labelled oxygen gases: Longitudinal assessment of cerebral oxygen metabolism in a rat model of neonatal hypoxic-ischaemic encephalopathy

**DOI:** 10.1177/0271678X231220691

**Published:** 2023-12-19

**Authors:** Saeka Shimochi, Jukka Ihalainen, Vilhelmiina Parikka, Nobuyuki Kudomi, Tuula Tolvanen, Ari Hietanen, Esa Kokkomäki, Stefan Johansson, Masahiro Tsuji, Shigehiko Kanaya, Emrah Yatkin, Tove J Grönroos, Hidehiro Iida

**Affiliations:** 1 Turku PET Centre, University of Turku 176483 , Turku, Finland; 2 MediCity Research Laboratory, 8058 University of Turku, Turku, Finland; 3 Nara Institute of Science and Technology, Ikoma City, Japan; 4 Department of Medical Physics, Turku University Hospital, Turku, Finland; 5 Accelerator Laboratory, Turku PET Centre, Åbo Akademi University, Turku, Finland; 6 Department of Pediatrics and Adolescent Medicine, Turku University Hospital, Turku, Finland; 7 InFLAMES Research Flagship Center, 8058 University of Turku, Turku, Finland; 8 Department of Medical Physics, Faculty of Medicine, 12850 Kagawa University, Kagawa, Japan; 9 Department of Food and Nutrition, Kyoto Women's University, Kyoto, Japan; 10 Central Animal Laboratory, 8058 University of Turku, Turku, Finland

**Keywords:** Animal model, cerebral oxygen metabolism, hypoxic-ischaemic encephalopathy, neonates, positron emission tomography

## Abstract

Perinatal hypoxic-ischaemic encephalopathy (HIE) is the leading cause of irreversible brain damage resulting in serious neurological dysfunction among neonates. We evaluated the feasibility of positron emission tomography (PET) methodology with ^15^O-labelled gases without intravenous or tracheal cannulation for assessing temporal changes in cerebral blood flow (***CBF***) and cerebral metabolic rate for oxygen (***CMRO_2_***) in a neonatal HIE rat model. Sequential PET scans with spontaneous inhalation of ^15^O-gases mixed with isoflurane were performed over 14 days after the hypoxic-ischaemic insult in HIE pups and age-matched controls. ***CBF*** and ***CMRO_2_*** in the injured hemispheres of HIE pups remarkably decreased 2 days after the insult, gradually recovering over 14 days in line with their increase found in healthy controls according to their natural maturation process. The magnitude of hemispheric tissue loss histologically measured after the last PET scan was significantly correlated with the decreases in ***CBF*** and ***CMRO_2._*** This fully non-invasive imaging strategy may be useful for monitoring damage progression in neonatal HIE and for evaluating potential therapeutic outcomes.

## Introduction

Perinatal asphyxia resulting in hypoxic-ischaemic encephalopathy (HIE) is a major cause of premature mortality worldwide, and 25% of survivors suffer from long-term neurological and neurodevelopmental impairments such as cerebral palsy.^1^ The incident occurs in approximately 1.5 per 1000 live births in developed countries.^2^ The pathophysiology starts with primary energy failure during the hypoxic-ischaemic (HI) event, involving oxidative metabolism failure, cytotoxic oedema and excitotoxin accumulation. The subsequent secondary energy failure results in glutaminergic excitotoxicity, apoptosis and neuroinflammation.^3^ The only clinically feasible therapy for HIE neonates is hypothermia starting within 6 h post-incident, but not more than 50% of treated neonates achieve an improved outcome.^4–6^ The predominantly employed Rice–Vannucci neonatal rodent HIE model^7,8^ has provided extensive advances toward developing promising therapies that could enhance the neuroprotective effect of hypothermia.

Given the large variation in brain damage severity in this animal model,^9^ *in vivo* follow-up observations on an individual basis are vital for consistent progress monitoring. This interindividual variability is also the case clinically in patients and thus reliable prognostic indicators are highly desired, particularly in the acute phase. In neonates, cerebral oxidative metabolism exhibits a distinct elevation in the early maturational process.^10,11^ Oxygen supply derangement to the brain at this stage accordingly causes devastating outcomes including reperfusion-related injury after the initial decrease in cerebral blood flow (***CBF***) post-HI incident.^12,13^ The subsequent progressive brain damage or healing process may be detected by a direct physiological parameter for brain tissue viability, i.e. the cerebral metabolic rate for oxygen (***CMRO_2_***). Thus, longitudinal monitoring of ***CBF*** and ***CMRO_2_*** in neonatal patients with their growth may aid in robustly assessing the disease course and potential treatment outcomes in association with normal developmental changes in brain energy metabolism. Moreover, this experimental approach should be minimally invasive, particularly for vulnerable neonatal rodents.

Studies have utilized the blood oxygenation level-dependent (BOLD) technique^14,15^ to quantitatively assess blood oxygenation level in the superior sagittal sinus region in humans using magnetic resonance imaging, from which the oxygen extraction fraction (***OEF***) can be accurately quantified for the whole brain. A combination of BOLD with blood velocity assessment using the phase-contrast technique in the carotid artery region provides ***CBF*** and thus ***CMRO_2_*** for the whole cerebral region.^16,17^ However, regional assessment or parametric imaging of ***OEF*** and ***CMRO_2_*** remains uncertain because of unknown signal behaviour in the diseased brain due to a varied vasculature abnormality that may violate the essential assumptions required in the theoretical model of Yablonskiy.^18^

Positron emission tomography (PET) with ^15^O-labelled gases has been the gold standard tool for decades for quantitative measurements of ***CBF***, ***CMRO_2_*** and ***OEF***. Despite the proven clinical value of ^15^O-PET, its application to small animals requires exceptionally intensive procedures like tracheotomy and repeated arterial blood sampling^19,20^ or the use of ^15^O-oxygen–labelled haemoglobin.^21,22^ The complexity of the technical procedure and kinetic modelling approaches that require correction for recirculating ^15^O-H_2_O are additional burdens. Recently, Temma et al. demonstrated a ^15^O-PET method that enables spontaneous inhalation of ^15^O-labelled gases without needing tracheotomy in mice.^23^ The technique enabled quantitative evaluations of ***CBF***, ***CMRO_2_*** and ***OEF*** over a one-month time course in a mouse model of chronic cerebral hypoperfusion with bilateral common carotid artery stenosis. Currently, no other methodology allows a non-invasive, repetitive and fully quantitative assessment of cerebral metabolic variables together with tissue perfusion in the 3D domain in experimental settings.

This study depicted temporal changes in ***CBF***, ***CMRO_2_*** and ***OEF*** after HI insult in rat neonates. We first assembled a novel integrated PET imaging system that enables sequential assessment of ***CBF***, ***CMRO_2_*** and ***OEF*** in rat neonates with spontaneous inhalation of ^15^O-labelled oxygen (^15^O-O_2_), carbon dioxide (^15^O-CO_2_) and carbon monoxide (^15^O-CO) gases. We also examined the ageing variation in those parametric values in healthy controls at term-equivalent ages to define a comparable time course of normal postnatal brain development across HIE pups.

## Materials and methods

### Study design

An integrated ^15^O-PET system was developed enabling quantitative ***CBF***, ***CMRO_2_*** and ***OEF*** assessment in small animals by spontaneous inhalation of ^15^O-O_2_, ^15^O-CO_2_ and ^15^O-CO. Using this system, two sets of experiments were performed; the first experiment was to determine the production rate of metabolized ^15^O-H_2_O (***k_w_***) in arterial blood after ^15^O-O_2_ inhalation and the second to assess the temporal changes in quantitative ***CBF***, ***CMRO_2_*** and ***OEF*** values in neonatal rats after HI insult compared with age-matched healthy or sham-operated controls.

### Animal subjects and ethical statement

For the first experiment that determined the ***k_w_***, thirteen Sprague–Dawley rats consisting of six rats (five males and one female) at 40.3 ± 0.8 days old (d/o) with a body weight of 155.0 ± 30.1 g and seven (all males) at 25.7 ± 1.0 d/o with 65.6 ± 1.0 g were studied. For the second experiment, nine male and nine female Sprague–Dawley rat pups at 9 d/o, whose brain developmental stage is considered equivalent to that of human infants at birth,^24,25^ from two litters were randomly divided into HIE, non-operated and sham groups, as summarized in Table 1.

**Table 1.** Age, body weight and number of animal subjects on each PET imaging day in the second experiment. Numbers in parentheses denote values after exclusion from the image data analysis.

PET scan day after HI insult	Day 0	Day 1	Day 2	Day 7	Day 14
Age (days old)	9	10	11	16	23
HIE					
Weights (gram)	24.0 ± 1.0(23.9 ± 1.1)	23.1 ± 2.1	26.1 ± 2.7	36.1 ± 4.5(35.3 ± 3.6)	50.6 ± 3.3
Animal number (n)	n = 5 (4)	n = 10	n = 11	n = 10 (7)	n = 5
Scan group 1	n = 5	n = 5	n = 6	n = 5 (2)	–
Scan group 2	–	n = 5	n = 5	n = 5	n = 5
Non-operated					
Weights (gram)	24.0 ± 2.9	–	27.8 ± 1.4	35.5 ± 3.8	54.4 ± 6.1
Animal number (n)	n = 4	–	n = 3	n = 4	n = 4
Sham					
Weights (gram)	–	–	–	40.2 ± 1.9	–
Animal number (n)	–	–	–	n = 3	–

HI: hypoxic-ischaemic; HIE: hypoxic-ischaemic encephalopathy; PET: positron emission tomography.

All animals were group housed under standard conditions (12-h light/dark cycle, temperature 21 ± 3 °C, humidity 55 ± 15%) with *ad libitum* access to RM3(E) rodent diet (SDS, UK) and tap water at the Central Animal Laboratory, University of Turku, Finland (UTUCAL). The study was approved by the National Project Authorization Board of Finland (ESAVI/20863/2018) adhering to the 3 R principles in accordance with Finnish National legislation (Act 497/2013, Decree 564/2013) and EU Directive 2010/EU/63 on the protection of animals used for scientific purposes. All reported experiments complied with the ARRIVE guidelines.^26^

### Integrated ^15^O-PET imaging system

A comprehensive PET system was assembled to sequentially assess ***CBF***, ***CMRO_2_*** and ***OEF*** in small animals as an extension of previous work.^23^ The entire system consisted of an ^15^O-oxygen–dedicated small cyclotron (C3D, IBA solutions, Louvain, Belgium), radiosynthesis and purification unit, gas chromatography for quality control, inhalation controller, dedicated PET scanners for each experiment and a radioactive gas evacuation system for effective scavenging of radioactive gases from the animal holder and radioactivity decay.

#### Radioactive gas production, purification/qualification and inhalation control

**Figure 1.**
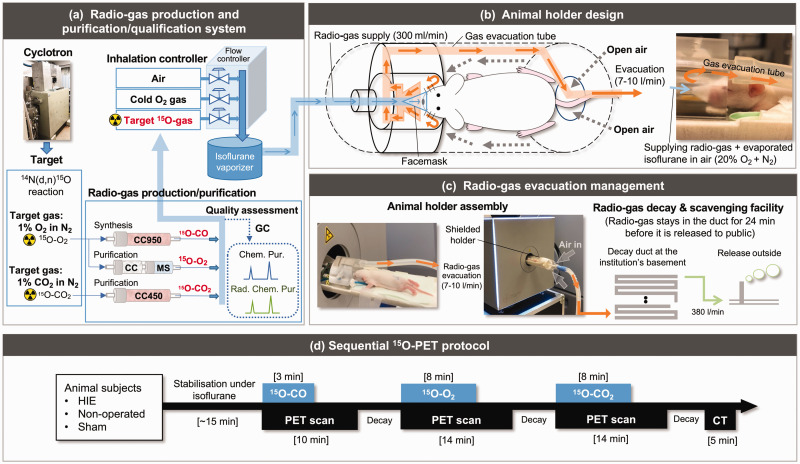
A schematic of the comprehensive PET system newly assembled in this study and consisting of three parts (a–c). (a) Radioactive gas production and purification/qualification system consisting of a cyclotron, target chamber, radioactive gas production/purification unit and inhalation controller. A charcoal column heated at 950 °C (CC950) was utilized for ^15^O-CO synthesis. For ^15^O-O_2_ purification, two columns filled with charcoal at room temperature (CC) and another with MS were used. For the purification of ^15^O-CO_2_, a column with charcoal heated at 450 °C (CC450) was used. Gas chromatography (GC) was implemented with two columns with HayeSep Q and Molecular sieve (MS) to assess chemical and radiochemical purity. (b) Animal holder dedicated to ^15^O-oxygen inhalation PET. A corn-shaped facemask made of a thin fabric sheet is placed over the snout of the animal. Radioactive gases are carried to the facemask for spontaneous inhalation. The animal holder is well sealed and actively scavenged from the front surrounding the radioactive gas supply tube. Fresh air is passively carried from the back into the animal holder. (c) Radioactive gas evacuation system. Approximately 380 l/min of evacuation carries scavenged radioactive gases from the animal holder to the decay ducts installed in the institute basement. Approximately 24 min of transit time in the ducts allows for radioactivity decay by a factor of 4000 before it is released from the chimney to the outside and (d) the ^15^O-PET/CT protocol consists of three separate PET scans each with ^15^O-CO, ^15^O-CO_2_ and ^15^O-O_2_ inhalation conducted in a sequential PET/CT study in the three subject groups of HIE, non-operated and sham. CT: computed tomography; HIE: hypoxic-ischaemic encephalopathy; PET: positron emission tomography; Radio-gas: radioactive gas.

^15^O was produced using the cyclotron by a ^14^N(d,n)^15^O nuclear reaction. The target gas was 1.0% O_2_ in N_2_ and 1.0% CO_2_ in N_2_, producing ^15^O-O_2_ and ^15^O-CO_2_ gases in the target, respectively. Each gas was transported to the hot cell, where ^15^O-O_2_ and ^15^O-CO_2_ gases were purified. The ^15^O-CO gas was synthesized from the target ^15^O-O_2_ gas by a charcoal column heated at 950 °C. The radiochemical and chemical purity was assessed each time before inhalation by gas chromatography (Model GC-2014, Shimazu, Kyoto, Japan) with Molecular Sieve and HayeSep Q columns (Figure 1(a)).

The quality-assured radioactive gases were then mixed with pure oxygen and air so that animals inhaled 20% oxygen during the entire period when animals were in the holder, even while radioactive gases were supplied or when the radioactivity supply rate was changed. The typical flow rate was 300 ml/min and the radioactivity supply rate was 250, 150 and 300 MBq/min, corresponding to ^15^O-O_2_, ^15^O-CO_2_ and ^15^O-CO, respectively. Using multiple radioactivity detectors and mass flow controllers reliably controlled these parameters. The inhalation gas was mixed with evaporated isoflurane gas at a given concentration (typically 2–2.5%) before it was transferred to the animal holder.

#### Dedicated ^15^O-PET system animal holder

Two sets of animal holder assemblies were constructed to adapt to the HRRT PET scanner (CTI PET Systems, Knoxville, TN, USA) for the first experiment and the β-Cube PET scanner (Molecubes, Ghent, Belgium) for the second experiment. Both holders were sealed to prevent radioactive gas leakage. A corn-shaped facemask made of a fabric sheet (100 μm thickness) gently covered the snout to which the radioactive gases were supplied at a flow rate of 300 ml/min. The air inside the animal holder was evacuated at 7–10 l/min from the front through small holes surrounding the supply tube inside the animal holder. Fresh air was then passively carried to the animal holder from the tail to the front (Figure 1(b)).

#### Radioactive gas evacuation system

The scavenged radioactive gas from the animal holder was carried to 28 decay ducts (each 9.5 m long and 150 mm diameter, total volume of 4649 l) implemented in the facility building, allowing sufficient radioactivity decay before releasing it from the facility (Figure 1(c)).

### Animal preparation and experimental procedures

#### First experiment (assessment of metabolized ^15^O-H_2_O)

Each animal assigned for the first experiment was anaesthetized with isoflurane (1.5–3%), and the left common carotid artery was cannulated with a polyethylene tube (SP-31, outer diameter: 0.8 mm, inner diameter: 0.5 mm, Natsume Seisakusho Co., Ltd, Tokyo, Japan). The cannula was secured by tight ligation around the cannulated vessel with 5-0 silk sutures (Ethicon, NJ, USA) and flushed with heparinized 0.9% NaCl to prevent blood clotting.

The animal was then moved to the animal holder and placed in the HRRT PET scanner. After ensuring the stabilisation of the animal under isoflurane anaesthesia, a 6 min attenuation correction scan was conducted. Next, a 6 min dynamic PET scan with a 5 s frame duration was started 15 s before initiation of ^15^O-O_2_ inhalation. Arterial blood samples (0.1 ml each) were manually obtained 4–5 times at 30 s intervals after 30 s of ^15^O-O_2_ inhalation from the catheter placed in the carotid artery. The blood samples were immediately divided into two samples. One sample was quickly centrifuged and the plasma radioactivity concentration was measured using a BeWell-Q3 well counter (Molecular Imaging Labo, Suita City, Japan), and the other sample was used to determine the whole blood radioactivity concentration.

#### Second experiment (sequential ^15^O-PET imaging)

HIE was induced in 9 d/o rats as described previously.^7,8^ Briefly, under isoflurane anaesthesia (1.5–3%), the left common carotid artery was exposed, ligated (6-0 silk sutures: Ethicon, NJ, USA) and then completely cut with electrocautery. After resting with their dam for 60 min, pups were exposed to 120 min of hypoxia in a chamber containing 8% oxygen and 92% nitrogen (P360 ProOx, BioSpherix, NY, USA) at 36–37 °C rectal temperature. Pups were then returned to their dam again for recovery for over 60 min and kept in standard housing condition after sequential imaging studies. Sham pups underwent a similar surgical procedure without carotid artery ligation and hypoxic insult. Non-operated pups experienced no interventions.

PET and computed tomography (CT) scans were sequentially performed on eleven HIE, four non-operated and three sham pups using β-Cube and X-Cube small animal scanners (Molecubes). During all scans, the animal was placed on a heating pad to maintain its body temperature, and respiration rate was continuously monitored using a sensor pad placed underneath the animal’s chest.

After ensuring the stabilisation of the animal under isoflurane anaesthesia, a series of list-mode PET scans were acquired (Figure 1(d)). Two 14 min scans were started at the time of initiating continuous inhalation of both ^15^O-O_2_ and ^15^O-CO_2_ over 8 min. A separate 10 min scan was acquired from the time of initiating continuous inhalation of ^15^O-CO over 3 min on one HIE pup on days 1, 7 and 14 before the ^15^O-O_2_ and ^15^O-CO_2_ scans to assess cerebral blood flow (***CBV***). Thereafter, the holder containing the animal was moved to the CT scanner for attenuation correction. Ten-to-fifteen-minute breaks separated PET scans to change cyclotron settings and allow radioactivity decay in the animal’s body. Since the β-Cube PET scanner has a limited data transfer rate of 8 Mcps in the electric circuit, the radioactivity supply rate was adjusted not to exceed this limitation during each inhalation. After PET/CT scans, the animal was kept on a heating pad to recover from anaesthesia and returned to its regular housing.

These sequential scans were repeated four times, first within 6 h (day 0) and then at 24 h (day 1), 48 h (day 2) and 7 days (day 7) post-insult for HIE pups in scan group 1, and on days 1, 2, 7 and 14 post-insult for HIE pups in scan group 2. Age-matched non-operated pups were sequentially scanned four times at ages equivalent to those of HIE pups on days 0, 2, 7 and 14. Sham pups were scanned only on day 7 after the surgical intervention. The PET imaging time course with postoperative days and ages in the three subject groups is presented in Table 1. Image data analysis excluded animals that indicated poor radioactivity inhalation dose in the body during scanning or inconstant body position between ^15^O-O_2_ and ^15^O-CO_2_ scans.

### Data processing

In the first experiment, PET images were reconstructed to generate dynamic images with 12 × 5 s, 8 × 15 s and 6 × 30 s frames for 6 min using the 3D OSEM method with depth-of-interaction compensation.^27^ Whole blood time-activity curves were obtained from the left ventricular (LV) region in the reconstructed images.

Well-counter counts for the plasma and whole blood samples were normalized by acquisition time duration(s) and weights for each sample, followed by correction for radioactivity decay to the PET initiation time. They were further corrected for the cross-calibration factor determined using a 5 cm diameter and 10 cm long cylindrical phantom so that the well counter-based activity concentration for whole blood was equivalent to that of the LV values in the PET image, as we previously reported.^28^ Plasma counting rates were corrected for the plasma/whole blood radioactivity concentration ratio for ^15^O-H_2_O.^29^ The arterial blood concentration of ^15^O-O_2_ [Bq/ml] was then obtained by subtracting the ^15^O-H_2_O concentrations from the whole blood radioactivity concentrations for each sample.

In the second experiment, PET images were reconstructed to generate dynamic images with a 1 min frame duration for the entire period of 14 min using 3D OSEM with depth-of-interaction compensation for ^15^O-O_2_ and ^15^O-CO_2_ and 10 min for ^15^O-CO scans. Radioactivity decay correction was set uncorrected in the second experiment to avoid degradation of the pixel count precision in decay-corrected images for short-lived ^15^O radioisotopes, which occurs specifically in the β-Cube scanner.

### Data analysis

#### Determination of the production rate of metabolized ^15^O-H_2_O in arterial blood

For the first experiment, a volume of interest (VOI) was selected on the LV cavity region in the dynamic PET images obtained after 30 s continuous inhalation of ^15^O-O_2_ using Carimas 2.1 (Turku PET Centre, Turku, Finland). The total blood arterial input function (AIF), $A_{t}(t)$ , was determined by interpolating the LV time–activity curve (TAC), as previously validated.^28^ We evaluated the agreement between this and the well counter-derived whole blood radioactivity concentration curves in each animal experiment.

The following model formulation was employed to estimate the metabolized ^15^O-H_2_O curve, $A_{w}(t)$ , from the total blood AIF, $A_{t}(t)$ :^25^

(1a) $$A_{w}(t)=k_{w}\text{ · }A_{t}(t)⊗e^{-k_{w}\text{·}t}$$

and then the ^15^O-O_2_ AIF, $A_{o}(t)$ , as shown below:

(1b) $$A_{o}(t)=A_{t}(t)-A_{w}(t)$$

where $k_{w}$ [min^−1^] is the production rate of the metabolized ^15^O-H_2_O in arterial blood after ^15^O-O_2_ inhalation. Next, we determined $k_{w}$ for each PET scan so that the simulated $A_{w}(t)$ in equation (1a) reproduced the well counter-derived metabolized ^15^O-H_2_O curve by means of non-linear least square fitting (NLLSF). $A_{w}(t)$ and $A_{o}(t)$ in the second experiment were then calculated from PET-derived $A_{t}(t)$ with the given k **_w_** following equations (1a) and (1b).

#### Calculation of parametric values

Parametric values of ***CBV***, ***CBF***, ***OEF*** and ***CMRO_2_*** were calculated for two VOIs selected in the brain. ***CBV*** *s* were first determined from a ^15^O-CO image for six examinations for both ipsi- and contralateral VOIs by normalising the average radioactivity concentrations in the cerebral VOIs by that in the LV VOI. The averaged ***CBV*** value was used in the following calculation.

***OEF*** and ***CBF*** (***f***) were both determined by simultaneous NLLSF of paired tissue time activity curves (tTACs) obtained with ^15^O-O_2_ and ^15^O-CO_2_ inhalation to the following equations.^29^

For the ^15^O-O_2_ inhalation PET data:

(2a) $$\begin{array}{c} C_{i}(t)=(1-V_{o})\cdot \mathrm{OEF}\cdot f\cdot A_{o}(t)⊗e^{-\frac{f}{p}\cdot t}\\ +(1-V_{w})\cdot f\cdot A_{w}(t)⊗e^{-\frac{f}{p}\cdot t}\\ +V_{o}\cdot A_{o}(t)+V_{w}\cdot A_{w}(t) \end{array}$$

and for the ^15^O-CO_2_ inhalation PET data:

(2b) $$C_{i}\left(t\right)=f\cdot A_{w}\left(t\right)⊗e^{-\frac{f}{p}\cdot t}+V_{w}\cdot A_{w}\left(t\right)$$

where the fractional volume of the arterial blood for ^15^O-O_2_ ( $V_{o}[\mathrm{ml}/\mathrm{ml}]$ ) and that for ^15^O-H_2_O ( $V_{w}[\mathrm{ml}/\mathrm{ml}]$ ) were determined from ***CBV*** [ml/ml] assuming a fractional venous blood volume (***F_vein_***) of 0.835^30^ as:

(3a) $$V_{o}=\mathrm{CBV}\cdot \{\left(1-F_{\mathrm{vein}}\right)+(1-\mathrm{OEF})\cdot F_{\mathrm{vein}}\}$$

(3b) $$V_{w}=\mathrm{CBV}\cdot (1-F_{\mathrm{vein}})$$

and ***p*** represents the tissue-to-blood partition coefficient of water (0.91 g/ml). The first-pass extraction fraction for ^15^O-water is limited in high flow regions, such as the rat brain, because of the high blood flow in small animals. Thus, this study defines ***CBF*** as a flux of ^15^O-water from the blood to the tissue space, as defined for ***CMRO_2_*** assessment using ^15^O-oxygen.

The AIF for ^15^O-O_2_ ( $A_{o}(t)$ ) and metabolized ^15^O-H_2_O ( $A_{w}(t)$ ) were estimated following equations (1a) and (1b), using the production rate of ***k_w_*** determined in experiment 1. The AIF for the ^15^O-CO_2_ inhalation experiment ( $A_{w}\left(t\right)$ ) was determined from the LV TAC as $A_{w}\left(t\right)=A_{t}\left(t\right)$ .

The ***CMRO_2_*** values were calculated as the product of ***CBF*** (***f***), ***OEF*** and the oxygen content in arterial blood, **[*O*_2_]_*a*_**, as:^30^

(4) $${\mathrm{CMRO}}_{2}={\left[O_{2}\right]}_{a}\times \mathrm{CBF}\times \mathrm{OEF}$$

where **[*O_2_*]_*a*_** is calculated as a product of the oxygen volume confined per unit gram of haemoglobin (1.39 [ml/g]), the haemoglobin concentration in the blood [g/ml] and the fractional saturation of oxygen in arterial blood (e.g. 98%).

#### Time course of parametric values

Time courses of absolute ***CBF*** (ml/min/g), ***CMRO_2_*** (ml/min/g) and ***OEF*** in ipsi- and contralateral brain VOIs were evaluated in the HIE, non-operated and sham groups over 14 days after the day of HI insult (day 0) or postnatal day 9. Age, group and hemispheric region dependency in ***CBF***, ***CMRO_2_*** and ***OEF*** were evaluated at each time point and between animal groups.

### Histological analysis

After the last PET scan on day 14, HIE and non-operated pups (n = 5 and n = 4, respectively) were transcardially fixed with 4% paraformaldehyde under deep anaesthesia with 4% isoflurane. The brains were removed, processed and embedded in paraffin sections. Coronal slices were obtained in 2 mm intervals from the frontal pole using a microtome (Leica RM2265, Nussloch, Germany) and stained with haematoxylin-eosin (H&E) for morphology. Microglial activation was examined using Iba1. Antigen retrieval was performed in citrate buffer (pH 6.0, BioSite BSC-OKHURL) for 20 min using a pressure cooker (Decloaking chamber, Biocare Medical NxGen). Sections were incubated with endogenous enzyme block (hydrogen peroxide) and pre-protein block (BrightDiluent, normal antibody diluent, WellMed BD09-125) to avoid non-specific binding. A primary antibody of Iba1 (1:2000, 019-19741, Wako Ltd., Japan) was applied for 60 min, followed by secondary antibody (BrightVision, 1 step detection system goat anti-rabbit HRP, WellMed DPVR110HRP) for 30 min and detection reagents (BrightDAB, WellMed BS04-110) for 10 min at room temperature.

The area (mm^2^) of each hemisphere was measured on H&E-stained sections using CaseViewer 2.4 (3DHISTECH Ltd., Budapest, Hungary). The hemispheric volume of each brain was calculated by summing the hemispheric area of each brain slice and multiplying the sum of the section thickness. The mean ipsi-/contralateral hemispheric-volume ratios were compared between HIE and non-operated groups. Additionally, the ipsi-/contralateral hemispheric-volume ratio in each HIE animal was compared with the ipsi-/contralateral ratios of ***CBF*** and ***CMRO_2_*** values obtained on day 14.

### Statistics

Results are presented as mean ± SD. The student’s paired or unpaired t-test was applied to compare two variables as appropriate. Pearson's analysis was used to test for correlation with GraphPad Prism 9 (GraphPad Software, San Diego, CA, USA). P-values <0.05 were considered statistically significant. Image data analysis was blinded to avoid any subjectivity.

## Results

Five of the thirteen rats that were assigned to the first experiment were excluded from the data analysis because of unsuccessful arterial blood sampling or extremely low ^15^O-O_2_ inhalation dose during the PET scan. Otherwise, all well counter-based whole-blood radioactivity concentrations agreed with the PET image-derived LV TAC in all cases and were further validated by an earlier study using the same experimental setup.^28^ Fitting the predicted metabolized ^15^O-water TAC to the manually separated ^15^O-water TAC resulted in average ***k_w_*** values of 0.33 ± 0.06, 0.33 ± 0.07 and 0.33 ± 0.07 $\;\min^{-1}$ , corresponding to younger (n = 4, 25.5 ± 1.0 d/o, 63.0 ± 0.8 g), older (n = 4, 40.0 ± 0.0 d/o, 166.5 ± 20.6 g) and all animals (n = 8, 32.8 ± 56.9 d/o, 114.8 ± 56.9 g), respectively. No significant difference was found in ***k_w_*** values between the two age groups. From these results, we defined a ***k_w_*** value of 0.33 min^−1^ in the calculation of ***CBF***, ***CMRO_2_*** and ***OEF*** for all animals in the second experiment.

**Figure 2.**
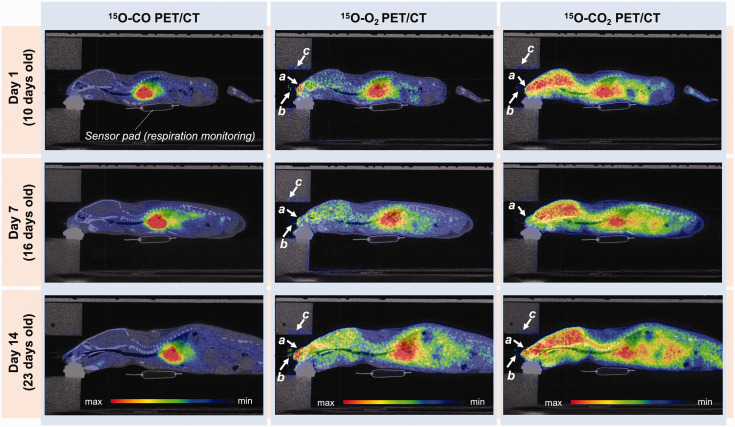
Representative sagittal whole-body PET/CT images of a HIE rat placed in the animal holder on days 1, 7 and 14 post-HI insult. Summed images over 0–10 min after ^15^O-CO, 0–5 min after ^15^O-O_2_ and 0–8 min after ^15^O-CO_2_ inhalation were displayed. White arrows (a–c) indicate the effects of radioactivity accumulated in the snout (a), emitted from the radioactive gas supply tube (b) and on the inner surface of the animal holder (c). CT: computed tomography; HIE: hypoxic-ischaemic encephalopathy; PET: positron emission tomography.

The whole-body distribution of ^15^O-gases in a rat scanned on days 1, 7 and 14 post-HI insult is shown in Figure 2 as sagittal PET/CT images accumulated for a period of 0–10 min, 0–5 min and 0–8 min after initiation of ^15^O-CO, ^15^O-O_2_ and ^15^O-CO_2_ gas inhalation, respectively. Radioactivity was visible on the surface of the snout inside the facemask (arrow a), in the gas supply tube (arrow b) and on the inner surface of the animal holder (arrow c). These radioactivity spots were negligibly small and did not significantly impact image data analysis. Radioactivity in the brain relative to the heart was lower with ^15^O-O_2_ than that with ^15^O-CO_2_. Radioactivity in the lung region was greater in ^15^O-O_2_ images than ^15^O-CO_2_ images.

**Figure 3.**
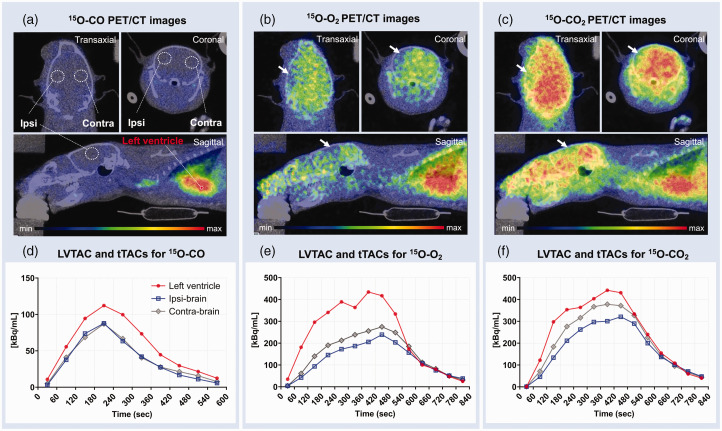
Representative transaxial, coronal and sagittal PET/CT images of the brain of a HIE rat on day 14 post-insult. PET images are summed over 0–10 min after ^15^O-CO (a), 0–5 min after ^15^O-O_2_ (b) and 0–8 min after ^15^O-CO_2_ (c) inhalation. The lesion is indicated by the white arrows in the left hemisphere in both ^15^O-O_2_ (b) and ^15^O-CO_2_ images (c). VOI locations in the LV, the lesion in the ligated-side (ipsilateral) hemisphere and its symmetrical position (contralateral) are also indicated on the ^15^O-CO images (a). The measured tTACs in ipsilateral (square) and contralateral (diamond) brain regions, and LV TAC (circle) from the same animal successively administered ^15^O-CO (d), ^15^O-O_2_ (e) and ^15^O-CO_2_ (f) are also presented. Note that LV TAC for ^15^O-CO is presented as one-tenth value (d). CT: computed tomography; HIE: hypoxic-ischaemic encephalopathy; LV, left ventricle; PET: positron emission tomography; TAC: time-activity curve; tTAC: tissue TAC; VOI: volume of interest.

Figure 3 shows ^15^O-gas accumulation in a HIE brain scanned on day 14 (Figure 3(a) to (c)) and LV TAC and tTACs (Figure 3(d) to (f)) for the selected VOIs defined in the ^15^O-CO image (Figure 3(a)). The decreased radioactivity in the ipsilateral hemisphere is clearly visible in both ^15^O-O_2_ and ^15^O-CO_2_ PET images (white arrows), while ^15^O-CO images showed no interhemispheric difference at any time point after HI insult. LV radioactivity concentrations were nearly equal between the ^15^O-O_2_ and ^15^O-CO_2_ inhalation scans although the supply rate of ^15^O-CO_2_ radioactivity was lower (200 MBq/ml) than that of ^15^O-O_2_ (500 MBq/ml). The peak values of TACs in both the ipsi- and contralateral brain regions were lower during ^15^O-O_2_ inhalation compared with ^15^O-CO_2_, as seen in Figure 3(b) and (c), respectively.

**Figure 4.**
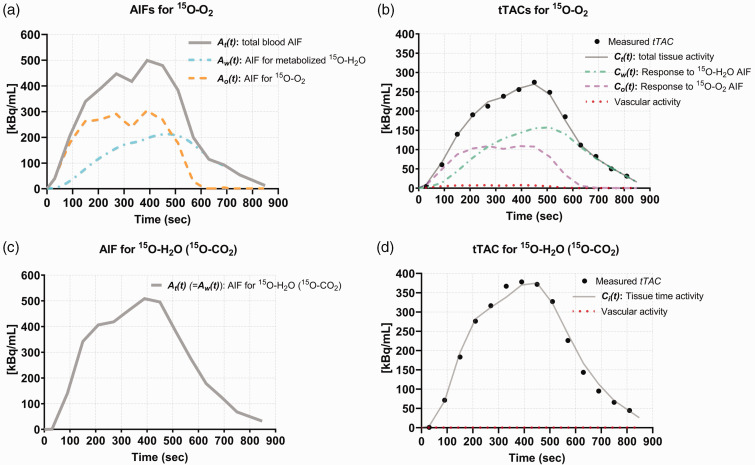
A typical example of AIFs (a) and the results from simultaneous fitting to tTACs (b) for ^15^O-O_2_, AIFs (c) and results from simultaneous fitting to ^15^O-CO_2_ tTACs (d) obtained from a HIE rat on day 14 after the insult (same case shown in Figure 3). (a) Three input functions are given for the ^15^O-O_2_ fitting analysis, i.e. the total blood AIF ( $A_{t}(t)$ ) (solid line), AIF for ^15^O-O_2_ ( $A_{o}(t)$ ) (dashed line) as calculated by equation (1b) and AIF for metabolized ^15^O-H_2_O ( $A_{w}(t)$ ) (dash-dotted line) as calculated by equation (1a) with the production rate of ^15^O-H_2_O, ***k_w_***, determined from the first experiment. The observed tTAC (closed circles) for the ^15^O-O_2_ scan was well reproduced by NLLSF (solid line) that consisted of the three components shown in (c), as estimated as a response to the ^15^O-O_2_ AIF (dashed line), a response to the AIF for ^15^O-CO_2_ (dash-dotted line) and the contribution of the blood radioactivity (dotted line), corresponding to the first, second and third terms of equation (2a), respectively. Of note is that the AIF for ^15^O-O_2_ reached to a plateau at approximately 3 min after the initiation of continuous inhalation of ^15^O-O_2_, while the contribution of metabolized ^15^O-H_2_O continued to increase, exceeding the contribution of ^15^O-O_2_ and reaching approximately 60% of the total radioactivity concentration at the end of ^15^O-O_2_ inhalation. In contrast, the AIF for ^15^O-CO_2_ inhalation consists of only a single component of ^15^O-H_2_O that is equal to the whole blood TAC, i.e. $C_{t}(t)$ . AIF: arterial input function; HIE: hypoxic-ischaemic encephalopathy; NLLSF: non-linear least square fitting; TAC: time-activity curve; tTAC: tissue TAC.

A representative result from the NLLSF analysis for fitting ***CBF*** and ***OEF*** simultaneously to two tTACs of ^15^O-O_2_ and ^15^O-CO_2_ is shown in Figure 4. Three AIFs were determined during ^15^O-O_2_ inhalation PET imaging from total blood ( $A_{t}\left(t\right)$ ) and metabolized ^15^O-H_2_O ( $A_{w}(t))$ and ^15^O-O_2_ ( $A_{o}(t)$ ), as estimated using equations (1a) and (1b), respectively (Figure 4(a)). $A_{t}\left(t\right)$ continued to increase during the ^15^O-O_2_ inhalation period of 8 min. However, $A_{o}(t)$ increased only until 3 min, plateauing at approximately 60% of the peak of $A_{t}\left(t\right)$ , while $A_{w}(t)$ increased until the end of ^15^O-O_2_ inhalation. Figure 4(b) demonstrates that the total tTAC ( $C_{t}\left(t\right)$ ) as given in equation (2a) reproduced well the measured tTAC. The additional three tTACs shown in this figure correspond to each of three terms shown in equation (2a), i.e. the response for ^15^O-O_2_ AIF ( $C_{o}\left(t\right)$ ), that for metabolized ^15^O-H_2_O AIF ( $C_{w}\left(t\right)$ ) and the vascular components ***V***_***o***_ · ***A*_*o*_** (***t***) + ***V_w_*** · ***A_w_*** (***t***). This figure also shows that while the total tTAC (***C_t_(t)***) continued to increase during the ^15^O-O_2_ inhalation period of 8 min, $C_{o}\left(t\right)$ plateaued at approximately 3 min until the end of ^15^O-O_2_ inhalation. In contrast, $C_{w}\left(t\right)$ exceeded the level of that for $C_{o}\left(t\right)$ around 4 min after ^15^O-O_2_ initiation. Figure 4(c) shows the AIF for ^15^O-CO_2_ inhalation, in which the total blood activity is equal to the AIF for ^15^O-H_2_O (***A_t_*(*t*) = *A_w_*(*t*)**), whereas Figure 4(d) presents the fit results for the ^15^O-CO_2_ inhalation scan given the formulation in equation (2b). The contribution of vascular radioactivity is negligibly small because of the small vascular component and large ***CBF*** values, as estimated in equation (3b). Averaged ***CBVs*** obtained from the ^15^O-CO scan were 0.049 ± 0.023 and 0.053 ± 0.026 ml/ml corresponding to the ipsi- and contralateral regions, respectively. Because of the small value, ***V_w_*** was neglected and the calculated ***V_o_*** was given as determined.

**Figure 5.**
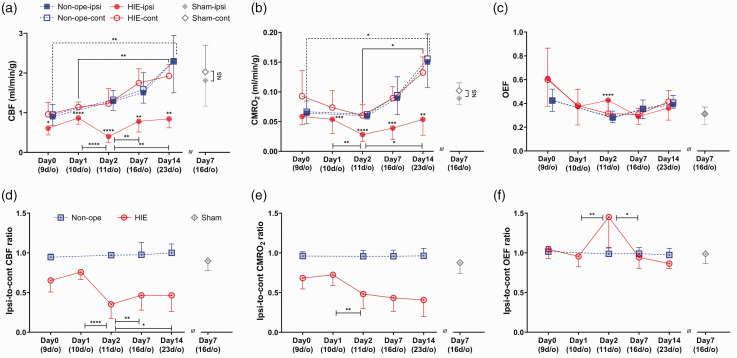
The mean temporal values of (a) ***CBF*** (ml/min/g), (b) ***CMRO_2_*** (ml/min/g) and (c) ***OEF*** in VOIs selected on ipsilateral (closed circle) and contralateral (open circle) hemispheres on days 0, 1, 2, 7 and 14 post-insult in HIE, at the corresponding age of 9, 11, 16 and 23 d/o in non-operated (ipsilateral: closed square; contralateral: open square) and on day 7 after the surgical operation in sham pups (ipsilateral: closed diamond; contralateral: open diamond). The ipsi- to contralateral ratios of ***CBF*** (d), ***CMRO_2_*** (e) and ***OEF*** (f) of the three subject groups on each imaging day (HIE: circle-dot; non-operated: square-dot; sham: diamond-dot) are also presented. Values are expressed as mean ± SD and those in a pair of each imaging time point or each hemisphere are compared by student’s paired t-test. Asterisks shown above closed circles indicate significant differences between each hemisphere. Ipsilateral HIE brains presented significantly lower values than contralateral hemispheres at all time points in ***CBF*** (a) and except for day 0 in ***CMRO_2_*** (b). ***OEF*** in ipsilateral HIE brains showed a significant increase on day 2 compared to contralateral hemispheres (c). *p < 0.05, **p < 0.01, ***p < 0.001, ****p < 0.0001, NS; not significant. CBF: cerebral blood flow; CMRO_2_: cerebral metabolic rate for oxygen; d/o: day old; OEF: oxygen extraction fraction; HIE: hypoxic-ischaemic encephalopathy; VOI: volume of interest.

The temporal profile of mean quantitative hemispheric ***CBF***, ***CMRO_2_*** and ***OEF*** values (Figure 5(a) to (c), respectively), and mean ipsi-/contralateral ratios of ***CBF***, ***CMRO_2_*** and ***OEF*** (Figure 5(d) to (f), respectively), as a function of days post-HI insult or equivalent days after birth (d/o) were determined in HIE, non-operated and sham subject groups (Figure 5). Non-operated pups showed significant increases from 9 to 23 d/o in both ***CBF*** (*p < *0.01) and ***CMRO_2_*** (*p < *0.05) by approximately 2.5-fold and 2-fold increases, respectively (Figure 5(a) and (b)). ***CBF*** in the ipsilateral brains of HIE pups was significantly reduced at the lowest level on day 2 (*p < *0.0001, compared to day 1) and then increased on day 7 (*p < *0.01) and day 14 (*p < *0.01) compared to day 2. ***CMRO_2_*** values in the ipsilateral hemispheres of HIE animals also reached the lowest levels on day 2 (*p < *0.01, compared to day 1) and significantly increased on day 14 from day 2 (*p < *0.05). Pearson’s test showed a correlation between age and ***CBF*** (*p = *0.0005) or ***CMRO_2_*** (*p = *0.0003) in non-operated pups. The ipsilateral hemispheres of HIE pups indicated a linear relationship between ***CBF*** (*p = *0.0007) or ***CMRO_2_*** (*p = *0.0125) and postoperative days from day 2 to day 14. ***OEF*** showed no time dependent or interhemispheric differences in any animal group except for a significant increase on day 2 in the ipsilateral hemisphere compared to contralateral hemisphere (*p < *0.0001) in HIE pups (Figure 5(c)). The ratios of ***CBF*** and ***CMRO_2_*** in HIE pups reached the lowest on day 2 and ***CMRO_2_*** ratios showed no temporal changes thereafter (Figure 5(d) and (e)). The ratio of ***OEF*** in HIE animals was significantly increased on day 2 (*p < *0.01, compared to day 1) and then decreased on day 7 (*p < *0.05) (Figure 5(f)).

**Figure 6.**
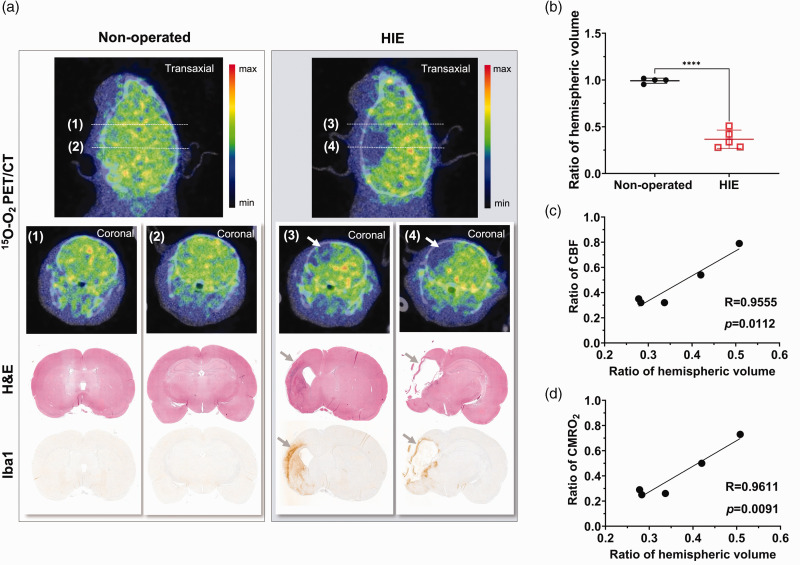
(a) Representative PET/CT images of the transaxial view and each coronal slice at the striatum (1) (3) and hippocampus level (2) (4) of the brains of a HIE pup on day 14 and an age-matched non-operated pup are shown as summed images over 0–5 min after ^15^O-O_2_ inhalation. Coronal images stained with H&E and Iba1 of the same animals at equivalent slicing levels are also displayed. Note that the area of the histological tissue loss in H&E images as indicated by grey arrows in the HIE animal is consistent with the defect region in ^15^O-O_2_ PET images as indicated by white arrows. Increased microglial activation is also visible around the infarct tissues in Iba1 images as indicated by grey arrows. (b) Ratios of the ipsilateral to contralateral hemispheric volume are presented as mean ± SD, displaying a significantly decreased mean ratio in HIE (****p < 0.0001) compared to non-operated pups (n = 5 and n = 4, respectively). (c, d) The ipsi-/contralateral ratio of ***CBF*** and ***CMRO_2_*** on day 14 in each HIE pup showed significant correlations with the ratio of the ipsi-/contralateral hemispheric volume in histology measured on the same day (*p = *0.0122 and *p = *0.0091, respectively). CBF: cerebral blood flow; CMRO_2_: cerebral metabolic rate for oxygen; CT: computed tomography; H&E: haematoxylin-eosin; HIE: hypoxic-ischaemic encephalopathy; PET: positron emission tomography.

A comparison of ^15^O-O_2_ PET/CT images and histology results from a HIE rat on day 14 and an age-matched non-operated rat is shown in Figure 6. Infarct regions measured by H&E staining in the HIE brain coincided with areas showing extremely decreased accumulation of ^15^O-O_2_ in PET images (Figure 6(a))_._ Additionally, increased microglial activation was displayed by Iba1 outside the margins of the infarct cores, where small reductions in ^15^O-O_2_ accumulation were observed. The mean ipsi-/contralateral hemispheric-volume ratio of HIE pups showed a 62.6% reduction compared with non-operated pups (*p < *0.0001) (Figure 6(b)). The hemispheric volume ratios of HIE pups indicated a linear relationship with the ratios of hemispheric ***CBF*** (*p = *0.0112) and ***CMRO_2_*** (*p = *0.0091) values measured in each brain VOI (Figure 6(c) and (d), respectively).

## Discussion

### Overview of the findings

The comprehensive ^15^O-oxygen PET system for small animals developed in this study provided quantitative ***CBF***, ***CMRO_2_*** and ***OEF*** values non-invasively by spontaneous inhalation of ^15^O-O_2_ and ^15^O-CO_2_ without tracheotomy or intravascular cannulations required using an intravenous ^15^O-H_2_O administration approach. The sophisticated radioactive gas supply and scavenging systems implemented for two PET scanners enabled good quality dynamic PET images. The physiological model-based estimation of metabolized ^15^O-H_2_O in arterial blood was essential as it avoids labour-intensive procedures and minimizes uncertainties in ***A_w_*** (***t***) and ***A_o_*** (***t***) values when assessing individual blood samples with small volumes. Simultaneous NLLSF to a pair of dynamic PET images for ^15^O-O_2_ and ^15^O-CO_2_ enabled reliable estimation of parametric values. This system allowed repetitive measurements even on consecutive days initiated immediately after the HI insult in vulnerable infant rats.

The technique detected time-dependent changes in ***CBF***, ***CMRO_2_*** and ***OEF*** associated with postnatal age and post-HI injury state for different brain regions and subject categories. Important findings in this study are the following: (a) remarkable reductions in ***CBF*** and ***CMRO_2_*** on day 2 after HI insult; (b) continuous recoveries after day 2 in the affected hemisphere in parallel to the increases in the contralateral hemisphere and (c) similar increase rates in ***CBF*** and ***CMRO_2_*** with age-matched controls (Figure 5). The non-operated rats displayed a relatively large increase from 9 to 23 d/o, which was similar to a previous report demonstrating that whole brain ***CBF*** and ***CMRO_2_***, measured by H_2_ clearance and the arteriovenous difference in O_2_ content, dramatically increased in infant rats from 10 to 20 d/o.^31^ Our finding is also consistent with a clinical PET study that indicated 3–4 times lower oxygen consumption in term infants than adults as a reflection of brain maturation.^32^ This difference is considered attributed to the escalating cerebral energy demand compensating for the increased Na+ pump activity in neuronal cells^33,34^ for structural and functional maturational processes at the ages examined in this study.

We confirmed that both LV TAC for ^15^O-oxygen scan in the first experiment agreed with those obtained by the well counter, which is consistent with an earlier finding^28^ despite the smaller average animal size in this study. This may be attributed to the contribution of the myocardium surrounding the LV chamber, which has a high flow value, resulting in a similar tTAC in the myocardial wall as the LV blood TAC. We also expect a similar phenomenon in the second experiment, in which a high-resolution β-Cube scanner was employed. However, this remains a hypothesis requiring further verification.

In HIE animals, ***CBF*** in the ipsilateral hemispheres showed a transient increasing tendency on day 1 post-insult ***_,_*** which may be consistent with a previous report, indicating that hyperperfusion occurring 24 h after HI intervention measured by the laser speckle method could be associated with the more severe tissue damage outcome.^13^ The subsequently marked reductions in both ***CBF*** and ***CMRO_2_*** on day 2 may be attributed to the neuronal cell death and apoptosis reportedly maximized at 24–72 h after neonatal HI insult.^8,35,36^ The pathophysiological background of the continuous increases in ***CBF*** and ***CMRO_2_*** in the ipsilateral VOI after day 2 may also be an important finding. Of note is that the ipsi-/contralateral ratio of ***CMRO_2_*** in HIE brains showed no significant increase after day 2. Thus, one may hypothesize that the surviving neuronal cells in the injured region keep growing while the damaged tissues with no energy consumption persisted.

Histological tissue losses measured after the last PET scan showed spatial agreement with severely decreased ***CBF*** and ***CMRO_2_*** areas (Figure 6). In contrast, ***CMRO_2_*** reduction was milder in the area where increased microglial activation was detected by Iba1 surrounding the infarct core region. Post-ischaemic inflammation reportedly modulates the exacerbation of neural damage in neonatal HIE, further suggesting a unique immune response in the immature brain.^37,38^ The application of repetitive imaging techniques of ^15^O-gas inhalation PET along with neuroinflammation-targeting PET using 18 kDa translocator protein (TSPO) would be useful for evaluating the age- and time-dependent effects of microglia-mediated inflammatory response on delayed neuronal damage expansion.

### Methodological advantages

^15^O-O_2_ gas is the sole tracer that can trace the kinetics of oxygen molecules and oxygen metabolism. It was shown in both the first and second experiments that ^15^O-O_2_ is metabolized quickly in neonatal rats so that the metabolized ^15^O-H_2_O replaces ^15^O-O_2_ in arterial blood shortly after starting ^15^O-O_2_ inhalation, as typically seen in Figure 4(a). Since the ^15^O-H_2_O extraction rate to the brain in the capillary bed is over two-fold higher than that of ^15^O-O_2_, the contribution of metabolized ^15^O-H_2_O to the brain (the second term in equation (2a)) exceeds the level of that of the original ^15^O-O_2_ during the continuous inhalation of ^15^O-O_2_ (Figure 4(b)), at 3–5 min after ^15^O-O_2_ inhalation initiation. To compensate for the contribution of metabolized ^15^O-H_2_O, the additional administration of ^15^O-CO_2_ (equivalent to intravenous ^15^O-H_2_O) was applied as many PET methods that utilized ^15^O-O_2_ inhalation protocol.

Simultaneous NLLSF is considered to be a reliable approach for estimating both ***CMRO_2_*** and ***CBF*** defined in equations (2a) and (2b), respectively. Using a single rate constant, ***k_w_***, that takes into account the kinetics of ^15^O-H_2_O in parallel to that of ^15^O-O_2_ plays an important role in this study in terms of stabilizing NLLSF estimation. This approach detected small but significant increases in ***CMRO_2_*** during cognitive stimulation that increased ***CBF*** to a greater amount;^29^ however, another approach failed to detect an increase in ***CMRO_2_*** where the metabolized ^15^O-H_2_O was neglected.^39^

### Limitations

There are several limitations to this study. Firstly, the animal model used exhibits hemispheric HI injury generated by unilateral ligation of the common carotid artery with subsequent global hypoxic insult. Considering the impact of interhemispheric collateral circulation, the use of ipsi-/contralateral ratios of ***CBF*** and ***CMRO_2_*** may have been biased, especially in the acute phase. Secondly, anaesthesia is a potentially confounding factor in measuring cerebral haemodynamics since isoflurane can derange ***CBF*** autoregulation.^40^ Furthermore, isoflurane may possess neuroprotective properties, and thus its exposure time may be correlated with decreased brain infarction in the Rice–Vannucci model.^41^ Another anaesthetic agent may be a suitable replacement, especially for the study of cerebral haemodynamics or ischaemic disease. It should also be mentioned that limited vital monitoring was performed because of the difficulty in placing highly sensitive equipment in the limited space of the animal holder to detect stable signals from small pups. Moreover, the physiological condition is unlikely constant during the entire PET imaging protocol because of the long scan period, and systematic errors may be attributed to transient changes in ***CBF*** and/or ***CMRO_2_***.

A standardized ***CBV*** value determined from limited animal scans of a single rat was used to calculate ***OEF*** and ***CMRO_2_***. The contribution of ***CBV*** to ***OEF*** and ***CMRO_2_*** was small and around 1% in the present data both in the control and HIE VOIs, partly attributed to the observed higher ***CBF*** and ***CMRO_2_*** values than those in humans. However, ***CBV*** could be increased, for example, in chronic ischaemic lesions,^42^ during haemodynamically altered conditions,^43^ and even in neurodegenerative diseases.^44^ Kinetic fitting of ***V_o_*** and ***V_w_*** with ***CMRO_2_*** and ***CBF*** as proposed by previously^45^ is highly desired as an alternative not only to remove the ^15^O-CO scan but also to improve the accuracy by removing the need for assuming a fixed arterial-to-venous blood volume ratio.

A ***k_w_*** of 0.33 ± 0.07 min^−1^ was obtained in the first experiment for 24 and 40 d/o rats, whereas it was approximately twice in an earlier study on 7–8-week-old rats.^45^ One reason for this discrepancy could be the different tracer administration procedures, i.e. earlier study used intravenous injection of ^15^O-O_2_–labelled oxyhaemoglobin instead of gaseous ^15^O-oxygen. Further systematic investigations are warranted.

We applied the average ***k_w_*** value obtained from the first experiment to all analyses for the second experiment. To estimate the effects of ±20% variation in ***k_w_***, the ***CBF*** and ***CMRO_2_*** values were calculated by changing ***k_w_*** by ±20% using the tTAC obtained from the day 14 study, which was the worst situation because of the highest ***CBF*** and ***CMRO_2_*** values. The change in ***k_w_*** by +20% caused changes in ***CBF*** by −3.7% and −7.5%, whereas a change by −20% resulted in +0.0% and +2.9%, corresponding to the ipsilateral and contralateral hemispheres, respectively. Similarly, changes in ***k_w_*** by +20% and −20% caused changes in ***CMRO_2_*** by +8.4% and +3.1% and −4.1% and −1.0%, respectively, in the ipsilateral and contralateral hemispheres. The range of changes in ***CBF*** and ***CMRO_2_*** were not large compared with the range of the time courses shown in Figure 5. However, a more systematic error propagation study is planned for future work.

### Future implications

The present ^15^O-oxygen PET system can be further improved if the entire examination duration is shortened. A PET scanner with better counting rate performance is essential for employing a single PET scan protocol during the dual inhalation of ^15^O-O_2_ followed by ^15^O-CO_2_ with a short interval, as demonstrated previously.^46,47^ This protocol can reduce the total anaesthetisation time and minimize possible physiological changes during PET scans. The intrinsic vascular radioactivity correction could also be compensated without the need for an additional ^15^O-CO scan. Continued research in this study would be of great value to evaluate possible pharmacological interventions and the early prognostic value of ^15^O-PET for neonatal HIE with a larger sample size. Such technical innovations would also facilitate the broader use of ^15^O-PET in preclinical and clinical applications, including in neonatal patients.

It is also important to establish a technique that can determine the appropriate ***k_w_*** value for the given subject groups without the need for blood sampling. The indirect calorimeter would allow assessments of ***k_w_*** through whole-body oxygen metabolism by measuring O_2_ consumption and CO_2_ production, as demonstrated earlier.^45^ More sophisticated equipment, such as a ventilated chamber for small animals,^48,49^ may also be a possibility.

## Conclusions

We demonstrated that the present ^15^O-gas inhalation PET system quantitatively assesses temporal changes in ***CBF*** and ***CMRO_2_*** associated with acute cerebral perfusion derangement and tissue damage evolving 48 h after HI insult in rat neonates. The technique also depicts subsequent pathological progression over 14 days interrelated with escalating cerebral energy metabolism along with the maturation process. This completely non-invasive imaging strategy may be of value for developing early therapeutic interventions and their response monitoring in neonatal HIE with highly individualized *in vivo* follow-up.
